# Genome-wide association and replication study of anti-tuberculosis drugs-induced liver toxicity

**DOI:** 10.1186/s12864-016-3078-3

**Published:** 2016-09-26

**Authors:** Zelalem Petros, Ming-Ta Michael Lee, Atsushi Takahashi, Yanfei Zhang, Getnet Yimer, Abiy Habtewold, Wondwossen Amogne, Getachew Aderaye, Ina Schuppe-Koistinen, Taisei Mushiroda, Eyasu Makonnen, Michiaki Kubo, Eleni Aklillu

**Affiliations:** 1 Laboratory for International Alliance on Genomic Research, RIKEN Center for Integrative Medical Sciences, Yokohama, Japan; 2 Department of Pharmacology, School of Medicine, College of Health Sciences, Addis Ababa University, Addis Ababa, Ethiopia; 3 Laboratory for Statistical Analysis, RIKEN Center for Integrative Medical Sciences, Yokohama, Japan; 4 Department of Internal Medicine, School of Medicine, College of Health Sciences, Addis Ababa University, Addis Ababa, Ethiopia; 5 AstraZeneca R&D, Innovative Medicines Personalised Healthcare & Biomarkers, SciLifeLab, Stockholm, Sweden; 6 Laboratory for Pharmacogenomics, RIKEN Center for Integrative Medical Sciences, Yokohama, Japan; 7 Laboratory for Genotyping Development, RIKEN Center for Integrative Medical Sciences, Yokohama, Japan; 8 Division of Clinical Pharmacology, Department of Laboratory Medicine, Karolinska University Hospital Huddinge C1:68, Karolinska Institutet, SE-141 86 Stockholm, Sweden

**Keywords:** Anti-tuberculosis, Drug induced liver injury, Ethiopian, *FAM65B*, C6ORF32, GWAS, *AGBL4*, Hepatotoxicity, Africa, Tuberculosis

## Abstract

**Background:**

Drug-induced liver injury (DILI) is a well-recognized adverse event of anti tuberculosis drugs (ATD) possibly associated with genetic variations. The objective of this study was to perform genome-wide association study (GWAS) to identify genetic variants associated with the risk for ATD induced liver toxicity in Ethiopian patients.

**Result:**

Treatment-naïve newly diagnosed tuberculosis patients (*n* = 646) were enrolled prospectively and treated with rifampicin based short course anti-tuberculosis therapy. Whole genome genotyping was done using Illumina Omni Express Exome Bead Chip genotyping array with 951,117 single nucleotide polymorphisms (SNPs) on 48 DILI cases and 354 ATD tolerants. Replication study was carried out for 50 SNPs with the lowest *P*-values (top SNPs) using an independent cohort consisting of 27 DILI cases and 217 ATD tolerants. In the combined analysis, the top SNP identified was rs10946737 (*P* = 4.4 × 10^−6^, OR = 3.4, 95 % confidence interval = 2.2–5.3) in the intron of *FAM65B* in chromosome 6. In addition, we identified a cluster of SNPs with suggestive genome-wide significance in the intron of ATP/GTP binding protein-like 4 (*AGBL4*).

**Conclusion:**

We identified genetic variants that are potentially associated with ATD induced liver toxicity. Further studies with larger sample sizes are essential to confirm the findings.

**Electronic supplementary material:**

The online version of this article (doi:10.1186/s12864-016-3078-3) contains supplementary material, which is available to authorized users.

## Background

Liver toxicity associated with drug treatment, known as drug-induced liver injury (DILI) is implicated in most cases of acute liver failure [1]. It can limit patient access to drugs that might otherwise be beneficial [2]. DILI is a major adverse event that leads to termination of clinical drug development programs and regulatory measures on approved drugs [3]. The largest population-based study reported on the incidence of DILI was from Iceland with a crude incidence rate of 19.1 cases per 100,000 inhabitants per year [4]. Although the causes of DILI can be various, studies have shown that genetic variations in genes involved in drug disposition, cellular stress, and immune response may contribute to DILI susceptibility [5, 6].

Anti-tuberculosis drugs (ATD) are among the most reported anti-microbial drugs incriminated to be potential causes of DILI [7]. ATD induced liver injury (ATDILI) is one of the most prevalent hepatotoxicities reported in many countries [8]. A previous study in Ethiopian tuberculosis (TB) patients showed 17.3 % incidence of ATDILI [9]. Incidence of treatment induced liver toxicity varies between populations. Higher incidence of concomitant ATD and antiretroviral (ARV) drugs induced liver toxicity in Ethiopian (30 %) compared to Tanzanian (10 %) TB and human immunodeficiency virus (HIV) coinfected patients has been reported [10, 11] Among the first line ATD, isoniazid, rifampicin, and pyrazinamide are known to cause DILI [8]. Genetic variations contribute to inter-individual ATDIL susceptibility [12]. Polymorphisms in drug metabolizing genes such as N-acetyltransferase 2 (*NAT2*), cytochrome P450 family 2 subfamily E polypeptide 1 (*CYP2E1*), glutathione S-transferase mu 1 (*GSTM1*), uridinediphosphate-glucuronosyltransferase1 family polypeptide A1 (*UGT1A1*) [8, 13], human leukocyte antigen (*HLA*) region [5, 8] and superoxide dismutase-2 mitochondrial (*SOD2*) gene [8, 14, 15] have been suggested to play roles in ATDILI.

Sub-Saharan Africa is disproportionally affected by high burden of TB and HIV. According to the latest WHO report, Ethiopia is listed among the top ten high-TB burden countries globally and one of the high multidrug resistant TB (MDR-TB) burden countries [16]. DILI is one of the important adverse events of anti-TB drugs, particularly during the intensive phase of TB therapy [17]. Treatment has to be discontinued in those patients who developed severe ATD induced liver toxicity, and treatment interruption may increase the risk for emergence of multidrug-resistant TB. Increased risk of developing MDR-TB in Ethiopian TB patients who encountered adverse events during the first course of TB treatment is reported recently [18]. Thus identification of genetic markers that predispose patients for ATD induced liver toxicity using GWAS in high TB burden sub-Saharan African countries, such as Ethiopia is imperative.

Using candidate gene approach, we previously identified genetic variation in *NAT2*, *CYP2B6*, and *ABCB1* genes as risk factors for ATD and antiretroviral (ARV) drugs co-treatment induced liver toxicity in TB-HIV co-infected patients [10, 11, 19]. Although candidate gene studies contribute to the discovery of genetic risk variants associated with ATDILI, the discovered genetic factors may account only for a proportion of the genetic variations, and some of the studies led to inconsistent results [20–22]. Therefore, we aimed to identify additional genetic variants through genome-wide association study (GWAS) for ATDILI in Ethiopian TB patients.

## Results

A total of 646 TB patients participated in this study and 75 (11.6 %) of them met the criteria for DILI diagnosis while on ATD treatment. Whole genome genotyping was done using genomic DNA from 48 DILI cases and 354 ATD tolerants. Replication study for 50 SNPs with lowest *P*-values (top SNPs) was done using genomic DNA from an independent cohort consisting of 27 DILI cases and 217ATD tolerants. The difference between the GWAS and the replication cohorts was based on time of first presentation. The first groups of patients were used for the GWAS, and the subsequent group of patients used for the replication study. The study area, TB diagnostic methods, case definitions, inclusion and exclusion criteria, and ATD treatment regimens used were all the same. The demographics and clinical characteristics of the study participants are presented in Table 1. There were statistically significant differences (*P* < 0.05) in HIV status and liver function test values between cases and treatment tolerants in both the GWAS and replication cohorts. There were statistically significant differences in sex, CD4 count and viral load between cases and treatment tolerants in the GWAS cohort but not in the replication study, which may be attributed to the smaller sample size of the replication cohort. More than one-third of the cases in our study had cholestatic pattern of DILI, and the rest had hepatocellular or mixed pattern.

**Table 1:** Demographics and clinical variables of the study participants

Variables	GWAS			Replication study		
	DILI Cases	Treatment tolerants	*P*	DILI cases	Treatment tolerants	*P*
No. of patients	48	354	-	27	217	-
Sex (M, F)	19, 29	203, 151	0.02	12, 15	85, 132	0.60
Age (yr), M (SD)	35.6 (10.4)	35.7 (11.5)	0.93	32.0 (7.4)	33.4 (10.3)	0.48
BMI (kg/m^2^), M (SD)	19.0 (3.2)	19.3 (3.0)	0.55	17.5 (3.0)	18.9 (3.0)	0.02
HIV positive, N (%)	44 (91.7)	225 (63.6)	<0.01	25 (92.6)	158 (72.8)	0.03
CD4 count, M (SD)	96.6 (78.5)	129.3 (120.8)	0.03	116.8 (98.3)	138.2 (121.0)	0.33
Viral load, log M (SD)	5.3 (0.9)	4.9 (0.9)	0.03	5.0 (0.8)	4.9 (0.9)	0.54
ALT (U/L), M (SD)	69.7 (37.2)	30.4 (14.4)	<0.01	67.2 (42.1)	30.7 (14.1)	<0.01
AST (U/L), M (SD)	101.2 (52.7)	40.5 (16.2)	<0.01	103.6 (71.9)	38.7 (13.6)	<0.01
ALP (U/L), M (SD)	187.7 (72.2)	121.1 (51.7)	<0.01	225.8 (139.9)	114.0 (63.1)	<0.01
T Bil (mg/dL), M (SD)	1.2 (1.0)	0.6 (0.4)	<0.01	1.1 (0.7)	0.5 (0.3)	<0.01
DILI pattern, N (%)						
Cholestatic	19 (39.6)			15 (55.6)		
Hepatocellular	10 (20.8)			5 (18.5)		
Mixed	19 (39.6)			7 (25.9)		

*ALP* Alkaline phosphatase, *ALT* Alanine aminotransferase, *AST* aspartate aminotransferase, *BMI* Body mass index, *DILI* Drug induced liver injury, *GWAS* Genome wide association study, *HIV* Human immunodeficiency virus, *M (SD)* Mean (standard deviation), *N* Number, *P* - *P* values, *T Bil* Total bilirubin

**Fig. 1:**
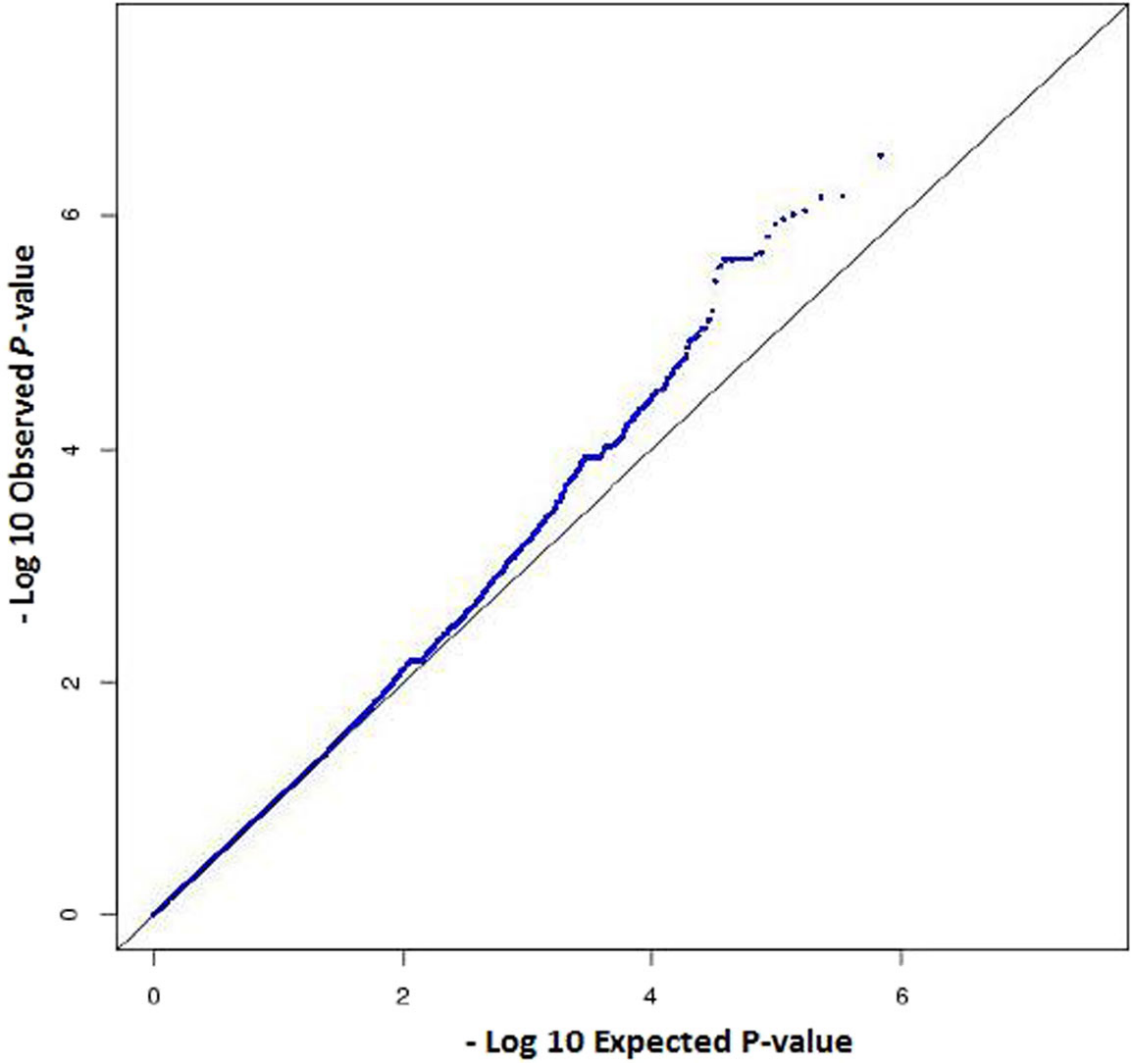
Quantile-quantile (QQ) plot for the observed versus expected *P*-values in trend test (λ_GC_ = 1.00007)

**Fig. 2:**
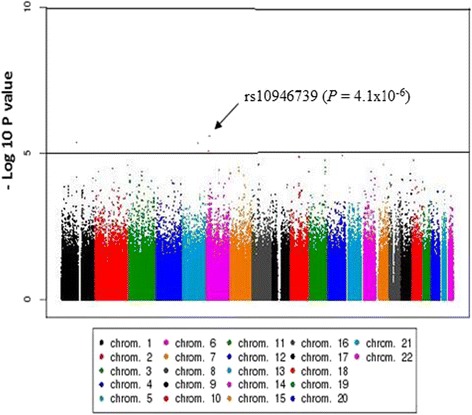
−Log_10_ *P* values of logistic regression across chromosomes in the GWAS

The Quantile-quantile (QQ) plot for the observed versus expected *P*-values, and Manhattan plot for the regression analysis are shown in Figs. 1 and 2, respectively. The top SNP in the GWAS after adjustment for sex, HIV status, CD4 count and HIV viral load was rs10946739 (*P* = 4.1 × 10^−6^, odds ratio (OR) = 3.4, 95 % CI = 2.0–5.6) located in the intron region of family with sequence similarity 65 member B (*FAM65B*), which is also named as chromosome 6 open reading frame 32 (*C6ORF32*) (Additional file 1: Table S1). The top SNP in the replication study after adjustment for covariates was rs319952 (*P* = 1.0 × 10^−2^, OR = 2.3, 95 % CI = 1.2–4.4) located in the intron of ATP/GTP binding protein-like 4 (*AGBL4*) in chromosome 1 (Additional file 1: Table S2). In the combined analysis, the top SNP after adjustment for covariates was rs10946737 (*P* = 4.4 × 10^−6^, OR = 3.4, 95 % CI = 2.2–5.3) located in the intron region of *FAM65B* (Table 2). In addition, four of the top SNPs (rs320035, rs393994, rs319952 and rs320003) were clustered in the intron of *AGBL4*.

**Table 2:** Top SNPs in the combined analysis of the GWAS and the replication study

SNP	Chr (loci)	Alleles (RA)	Study	Cases/controls	MAF	*P*_min	*P*_adj	OR (95 % CI)	Nearest gene
rs10946737	6 (24967240)	A/G (A)	GWAS	48/354	0.10	2.0 × 10^−5^	9.7 × 10^−6^	4.3 (2.5–7.4)	*FAM65B*
			Rep	27/216	0.10	3.8 × 10^−2^	8.6 × 10^−2^	2.2 (0.9–5.4)	
			Comb	75/570	0.10	6.3 × 10^−7^	4.4 × 10^−6^	3.4 (2.2–5.3)	
rs320035	1 (49089197)	A/G (G)	GWAS	48/354	0.48	3.5 × 10^−6^	1.3 × 10^−4^	2.4 (1.5–3.8)	*AGBL4*
			Rep	27/216	0.50	4.2 × 10^−3^	1.2 × 10^−2^	2.2 (1.9–3.9)	
			Comb	75/570	0.49	8.2 × 10^−7^	5.1 × 10^−6^	2.3 (1.6–3.3)	
rs10946739	6 (24993127)	A/G (A)	GWAS	48/354	0.19	9.6 × 10^−6^	4.1 × 10^−6^	3.4 (2.0–5.6)	*FAM65B*
			Rep	25/209	0.18	1.1 × 10^−1^	1.8 × 10^−1^	1.7 (0.8–3.6)	
			Comb	73/563	0.19	4.7 × 10^−6^	5.1 × 10^−6^	2.7 (1.8–4.1)	
rs393994	1 (49108745)	T/C (C)	GWAS	48/354	0.48	6.1 × 10^−6^	1.7 × 10^−4^	2.4 (1.5–3.7)	*AGBL4*
			Rep	27/216	0.50	7.9 × 10^−3^	1.4 × 10^−2^	2.1 (1.2–4.0)	
			Comb	75/570	0.49	1.9 × 10^−6^	7.6 × 10^−6^	2.3 (1.6–3.3)	
rs320003	1 (49126778)	A/G (A)	GWAS	48/354	0.48	1.7 × 10^−5^	2.3 × 10^−4^	2.3 (1.5–3.7)	*AGBL4*
			Rep	23/208	0.50	1.9 × 10^−2^	1.2 × 10^−2^	2.3 (1.2–4.5)	
			Comb	71/562	0.49	4.6 × 10^−6^	8.3 × 10^−6^	2.3 (1.6–3.4)	
rs319952	1 (49113622)	A/G (G)	GWAS	48/354	0.48	1.1 × 10^−5^	2.8 × 10^−4^	2.3 (1.5–3.6)	*AGBL4*
			Rep	26/216	0.50	1.2 × 10^−2^	1.0 × 10^−2^	2.3 (1.2–4.4)	
			Comb	74/570	0.49	2.5 × 10^−6^	8.5 × 10^−6^	2.3 (1.6–3.3)	
rs7958375	1 (2 111640017)	A/G (A)	GWAS	48/354	0.02	8.8 × 10^−5^	1.2 × 10^−5^	11.3 (3.8–33.5)	*CUX2*
			Rep	27/216	0.02	1.0 × 10^+0^	7.4 × 10^−1^	1.5 (0.2–13.1)	
			Comb	75/570	0.02	1.7 × 10^−4^	4.6 × 10^−5^	7.6 (2.9–20.0)	

*Chr (loci)* Chromosome, and chromosomal loci based on NCBI built 37, *CI* Confidence Interval, *Comb* Combined analysis using inverse variance method, *GWAS* Genome wide association study, *MAF* Minor allele frequency, *OR* Odds ratio, *P_adj* Logistic *P*-value after adjustment for sex, HIV status, CD4 count and HIV viral load; *P_min* Minimum *P*-value among allelic, dominant and recessive models of Fisher’s exact test, and *P*-value of inverse variance combined analysis; *RA* Risk allele, *Rep* Replication study, *SNP* Single nucleotide polymorphism

For the sub-group analysis based on the pattern of liver injury, the top SNPs for cholestatic, hepatocellular and mixed patterns of DILI were rs10182566 (*P* = 4.1 × 10^−6^, OR = 6.0, 95 % CI = 2.8–12.8) in 3′-untranslated region of chromosome 2 open reading frame 71 (*C2orf71*), rs1990046 (*P* = 3.7 × 10^−6^, OR = 28.4, 95 % CI = 6.9–117.3) in the intron of semaphorin3A (*SEMA3A*) in chromosome 7, and rs12603186 (*P* = 8.1 × 10^−6^, OR = 7.2, 95 % CI = 3.0–17.2) in shisa family member 6 (*SHISA6*) in chromosome 17, respectively (Additional file 1: Table S3).

## Discussion

In this study, we carried out GWAS and replication analysis on a total of 646 patients treated with ATD to identify novel genetic variants associated with DILI. Previously we investigated pharmacogenetic markers for concomitant ARV and ATD co-treatment induced DILI in TB-HIV co-infected patients (*n* = 353) using candidate gene approach [10]. As a continuation, we conducted a large prospective cohort study in 1060 patients, where we evaluated the patterns of ATD and/or ARV drugs induced liver toxicities [23]. In the present study, we investigated for possible genetic markers for ATD induced liver toxicity using genome wide association approach in 646 selected study participants from the recent large prospective cohort study by considering DILI cases developed during anti-TB treatment only. Identifying the risk variants could help developing clinical tests to prevent DILI, and to match the patients with alternative, effective and safe medications. To our knowledge, this is the first GWAS for ATDILI in an African population.

The top SNP in the GWAS analysis after adjustment for covariates was in the intron of *FAM65B*. This gene encodes a cytoplasmic protein that plays a role in myoblast differentiation, and it is transiently up-regulated during early stage of the process [24]. Alternative splicing of this gene results in multiple transcript variants. Inhibition of expression of this gene in myoblasts causes marked decrease in myogenin expression with consequent lack of myoblast fusion; and its over-expression induces formations of cellular protrusions [25]. It is suggested that *FAM65B* may possibly play a role in myoblast migration, and mutations could affect muscle development and human muscle diseases; however, its exact role is still largely unknown [25]. According to the human Protein Atlas data, *FAM65B* is also a mitochondrial protein expressed in the liver hepatocytes, gall bladder and bile duct [26]. Recent studies indicate that *FAM65B* plays a role in cancer and liver inflammation [27]. Further analysis is necessary to explain functional importance of *FAM65B* gene in ATDILI.

Strong association with ATDILI was identified by a cluster of four SNPs with *P*-values suggestive of genome-wide association significance in the intron of *AGBL4* (*CCP6*). This gene encodes an enzyme that catalyzes deglutamylation of polyglutamate side chains generated by post-translational modification of target proteins like tubulins in microtubules [28]. Further analysis is required to explain the role of *AGBL4* gene and its contribution to individual differences for susceptibility to ATDILI. The identified genetic risk variants in our study if replicated in larger sample sizes and in other populations, they may serve as genetic biomarkers for ATDILI.

It is increasingly evident that genetic variants can determine an individual’s susceptibility to develop a particular pattern of liver injury [29]. Therefore, we performed sub-group GWAS analysis based on the pattern of DILI. The SNP (rs1990046) with the smallest *P*-value after adjustment for covariates (*P* = 3.7 × 10^−6^) was identified in the hepatocellular type of DILI. This SNP is located in the intron region of *SEMA3A*, a member of the semaphorin family. This gene encodes a protein with an immunoglobulin-like domain and sema domain, which is vital for normal neuronal pattern development [30], and also plays a role in the pathogenesis of allergic conditions such as allergic rhinitis [31]. However, further studies are required to elucidate the role of *SEMA3A* gene in hepatocellular pattern of ATDILI.

In our previous candidate gene study [10], genetic variants in genes involved in drug metabolism of ATD like *NAT2* were associated with DILI. Variants in other drug metabolizing genes [8, 13], *HLA* region [5, 8], and in genes related to oxidative stress [32] and autoimmune diseases [2] were also reported to have association with susceptibility to ATDILI. In our GWAS, we did not find genetic variants that passed genome-wide significance in these genes, which may be related to the limited sample sizes used for the study. But we found possible association SNPs rs12969241 (*P* = 1.1 × 10^−5^) located in the intron region of protein tyrosine phosphatase non-receptor type 2 (*PTPN2*), rs2842997 (*P* = 5.1 × 10^−3^) in the vicinity of *SOD2*, and rs12543818 (*P* = 1.9 × 10^−3^) near *NAT2* for genes related to autoimmune diseases, oxidative stress and pharmacokinetics, respectively (Additional file 1: Table S4). The SNP rs12969241 in the *PTPN2* gene was also among the top in the GWAS of cholestatic pattern of DILI (*P* = 6.8 × 10^−6^) (Additional file 1: Table S3). The protein encoded by the *PTPN2* gene is an intracellular tyrosine-specific phosphatase, which is expressed in epithelial cells, fibroblasts or endothelial cells [33]. This protein was shown to play an important role in the protection of epithelial barrier function during inflammation by acting as negative regulator of pro-inflammatory cytokine interferon-γ [34]. This finding may indicate the implication of an immune related mechanism in ATDILI. The product of *SOD2* gene, which was identified for genes related to oxidative stress, detoxifies highly reactive superoxide radicals generated by mitochondrial respiration [35]. This finding is in line with a previous study [15], which reported common polymorphisms in *SOD2* as predictor of ATDILI. We speculate that ATDILI may be related to the combined effect of the new variants identified, pharmacokinetic, oxidative stress, and immune-related gene variants.

Sub-Saharan African population is the most genetically heterogeneous population globally, characterized by extensive population substructure, and less linkage disequilibrium (LD) among loci compared to non-African populations.[36]. Although GWAS in populations of African ancestry is challenging due to less degree of LD; the high level of genetic diversity and weak LD with neighboring SNPs in Africans ancestry is considered as a powerful tool for fine mapping causal variants that underlie common diseases or complex traits found globally [37]. The advantage of conducting GWAS in African ancestry populations in the context of addressing existing and emerging global health conditions is reported recently [37]. The present study exploring ATDILI risk variants through GWAS in Ethiopia, the second most densely populated country in Africa, will not only provide national genomic information for personalized medicine but also may contribute to the advancement of pharmacogenomics in Africa.

There were some limitations for this study. First, as the DILI cases are rare and were difficult to collect (four years were required to identify 75 ATDILI cases), this resulted in small number of case samples particularly for sub-group analysis based on the pattern of DILI. Second, populations of African ancestry, as in case of our study population, have greater genetic diversity and lower levels of linkage disequilibrium (LD) among chromosomal loci [38]. The low levels of LD are disadvantageous when screening the genome for disease associations using the current SNP-genotyping approaches that essentially rely on the principle of LD mapping. Therefore, additional studies with higher density SNP array or next generation sequencing may be required to discover susceptibility variants in such population. Ethiopian population display distinct pharmacogenetic variations compared to other black African population [39–42], and thus results from this study may not be directly extrapolated to other sub-Saharan African population. However, our exploratory study using homogenous well-characterized clinical samples for the discovery and replication of new DILI biomarkers, represents an important first step in applying GWAS to identify genetic variants for ATDILI. The third limitation is that the current protocol of TB treatment consists of combinations of drugs, thus we cannot affirm that the risk variants identified corresponds only to a single drug or multiple drugs in the treatment regimen.

## Conclusion

Using genome-wide wide associations study, we identified potential genetic variants associated with ATDILI. The results provide evidence that in addition to genetic variants identified by candidate gene studies, other variants also influence the risk of developing DILI by ATD. Further replication studies are essential to confirm the findings.

## Methods

### Study participants and treatment

The participants for the present GWAS were selected from a recent prospective cohort study where patterns of antiretroviral therapy (ART) and/or anti-TB treatment induced liver toxicity was investigated [23]. In brief newly diagnosed treatment naïve patients enrolled into one of the following study arms were considered for the present study:

1. TB infected patients (with out HIV co-infection) treated with rifampicin based ATD only.
2. TB-HIV co-infected patients with baseline CD4 count >200 cells/mm^3^ (not eligible for ART, following the national and WHO treatment guideline valid during the study period) and treated with rifampicin based ATD only.
3. TB-HIV co-infected patients with baseline CD4 count <200 cells/mm^3^ and (eligible for ART co-treatment) and rifampicin based ATD was initiated first followed by efavirenz based ART (delayed up to 8 weeks after starting ATD). Patients who developed DILI while on ATD treatment only (before starting ARV therapy) were included in the current GWAS, but patients who developed DILI after initiating ARV co-treatments were excluded from this study.

Patients were recruited from three health institutions: Kazanchis and Beletshachew health centers and Black Lion specialized referral and teaching university hospital in Addis Ababa, Ethiopia [23]. Diagnosis of TB was based on sputum smear, fine needle aspirate, clinical and radiological evidences. The eligibility criteria were TB confirmed men and non-pregnant women, age ≥18 years and receiving no other known hepatotoxic drugs concurrently. Patients who had abnormal liver enzyme biochemistry at baseline, positive serological test for either hepatitis B virus surface antigen or anti-hepatitis C virus antibody or known liver injury prior to starting treatment were excluded.. Written informed consent was obtained from all the study participants prior to study enrolment. The study protocol was approved by the Institutional Review Board of College of Health Sciences, Addis Ababa University, Ethiopia; Ethical Review Board of Karolinska Institutet, Sweden; and Ethical Review Committee of RIKEN, Japan.

Drug treatment was initiated according to World Health Organization (WHO) and Ethiopian National TB Treatment Guidelines as described previously [23]. All patients received short-course ATD treatment consisting of rifampicin (150 mg), isoniazid (75 mg), pyrazinamide (400 mg) and ethambutol (275 mg) for the first two months in fixed dose combinations given daily under direct observed therapy during the intensive phase, followed by rifampicin (150 mg) and isoniazid (75 mg) for the next four months in fixed dose combinations given daily. The treatment dosage was based on the weight of the patient: 20–29 kg (1½ tablets), 30–37 kg (2 tablets), 38–54 kg (3 tablets) and ≥55 kg (4 tablets). Liver function tests were carried out at baseline and on the 1st, 2nd, 4th, 8th, 12th and 24th weeks after initiation of treatment.

### Case definitions

For DILI case definitions, the criteria set by the International DILI expert working group were used [43]. The upper limit of normal (ULN) for liver biochemical parameters used for the study population were alanine aminotransferase (ALT 33 U/L, male; 29 U/L, female), aspartate aminotransferase (AST, 41 U/L), alkaline phosphatase (ALP, 128 U/L), and 1.0 mg/dL for total bilirubin [23]. All cases recruited met at least one of the following criteria: − (1) ALT ≥5xULN, (2) ALP ≥2xULN, or (3) ALT ≥3xULN along with total bilirubin ≥2xULN. All cases had a minimum score of three (‘possible’) in Roussel Uclaf Causality Assessment Method (RUCAM) scoring system for DILI. The pattern of liver injury was defined using R-values where, R = (ALT/ULN)/(ALP/ULN). Cases were categorized as having hepatocellular (*R* ≥ 5), cholestatic (*R* ≤ 2), or mixed (2 < R < 5) pattern of DILI [43]. Treatment tolerants for the study were defined as individuals who were also on short course ATD treatment but did not fulfill the case definitions for DILI, and had not presented clinical signs and symptoms consistent with DILI in the follow up period [23].

### Whole genome genotyping and quality control

Genomic DNA was isolated from whole blood samples using QIAamp DNA Maxi Kit (QIAGEN GmbH, Hilden, Germany). Genotyping was conducted in RIKEN Center for Integrative Medical Sciences, Yokohama, Japan. Whole genome genotyping was done using Illumina Omni Express Exome Bead Chip genotyping array (Illumina Inc., San Diego, CA, USA) according to the manufacturer’s protocol. This array captures 951,117 single nucleotide polymorphisms (SNPs). To further validate the results of the GWAS analysis, replication study was then carried out for 50 SNPs with the lowest *P*-values (top SNPs) using an independent cohort. Genotyping for the replication study was done using multiplex polymerase chain reaction (PCR) based Invader assay [44] with ABI PRISM 7900HT Sequence Detection System (Applied Biosystems, Foster City, CA, USA).

For data cleaning, systematic stepwise quality filtering of raw genotyping data was done using PLINK [45]. From an initial full set, those SNPs not mapped on autosomal chromosomes were filtered out. In addition, SNPs with a call rate less than 99 %, minor allele frequency less than 0.01, or deviated from expected Hardy-Weinberg equilibrium (*P* < 1.0 × 10^−6^) were removed. A total of 660, 206 SNPs that passed the quality filter were used for further analysis. Individuals were checked for gender concordance between recorded clinical data and genotype determined sex. Samples with genotyping call rate greater than 99 % were included in the analysis. Quantile-quantile plot comparing the expected and observed *P*-values was performed in R-statistical environment, and genomic control inflation factor (λ_GC_) was computed to detect population stratification [46].

#### Statistical analysis

After the quality filter, the tests of associations were done using PLINK v1.07 [45]. For each SNP, Fisher’s exact test using the three genetic inheritance models (dominant, recessive, allele frequency) were carried out to compare allele and genotype frequencies between DILI cases and treatment tolerants. SNPs were rank-ordered according to the minimum *P*-value in the genetic models. The threshold for genome-wide significance for associated SNPs was determined using Bonferroni correction (*P* < 7.6 × 10^−8^). SNPs with *P*-values below 10^−5^ were considered suggestive of genome-wide significance. Logistic regression analysis adjusted for sex, HIV status, CD4 count and HIV viral load as covariates was performed. These variables were associated with DILI as described previously [9, 10]. Combined analysis of GWAS and replication study was conducted using inverse-variance method [47]. Manhattan plot was generated using Haploview software to visualize the results [48]. We also performed sub-group GWAS analysis based on the pattern of liver injury.

## Additional file

Additional file 1: Table S1. Top fifteen SNPs in the GWAS of ATDILI. **Table S2.** Top fifteen SNPs in the replication study of ATDILI. **Table S3.** Top SNPs in the GWAS of the pattern of ATDILI. **Table S4.** Top SNPs for GWAS of ATDILI in genes related to autoimmune diseases, oxidative stress, pharmacokinetic, and HLA region. (PDF 57 kb)
