# Supplementary material for: Genome-wide association and replication study of anti-tuberculosis drugs-induced liver toxicity
Source: BMC Genomics. 2016 Sep 26;17:755. doi: 10.1186/s12864-016-3078-3 (PMC5037629; doi:10.1186/s12864-016-3078-3)
Supplement: Additional file 1: Table S1. — Top fifteen SNPs in the GWAS of ATDILI. Table S2. Top fifteen SNPs in the replication study of ATDILI. Table S3. Top SNPs in the GWAS of the pattern of ATDILI. Table S4. Top SNPs for GWAS of ATDILI in genes related to autoimmune diseases, oxidative stress, pharmacokinetic, and HLA region. (PDF 57 kb) [file 12864_2016_3078_MOESM1_ESM.pdf]

Table S1. Top fifteen SNPs in the GWAS of ATDILI.

SNP	Chr (loci)	Alleles (RA)	11vs	22vs	1vs2	*P*_min	*P*_adj	OR (95% CI)	MAF	Nearest gene
**rs10946739**	**6 (24993127)**	**A/G (A)**	$1.1x10^{-3}$	$2.5x10^{-4}$	$9.6x10^{-6}$	$9.6x10^{-6}$	$4.1x10^{-6}$	**3.4 (2.0-5.6)**	**0.19**	***FAM65B***
**rs7708937**	**5 (123169574)**	**T/C (T)**	$4.0x10^{-2}$	$1.1x10^{-5}$	$4.8x10^{-6}$	$4.8x10^{-6}$	$4.4x10^{-6}$	**4.0 (2.4-6.7)**	**0.11**	***KRT18P16***
**rs10946737**	**6 (24967240)**	**A/G (A)**	$1.2x10^{-1}$	$2.4x10^{-5}$	$2.0x10^{-5}$	$2.0x10^{-5}$	$9.7x10^{-6}$	**4.3 (2.5-7.4)**	**0.10**	***FAM65B***
**rs2089910**	**11 (1874404)**	**T/C (T)**	$1.2x10^{-2}$	$2.2x10^{-5}$	$5.3x10^{-6}$	$2.2x10^{-6}$	$4.2x10^{-5}$	**2.7 (1.8-4.2)**	**0.30**	***LSP1***
**rs4903067**	**14 (73286300)**	**T/C (C)**	$9.9x10^{-6}$	$2.9x10^{-1}$	$6.1x10^{-5}$	$9.9x10^{-6}$	$8.1x10^{-5}$	**3.0 (1.7-5.1)**	**0.17**	***DPF3***
**rs320035**	**1 (49089197)**	**A/G (G)**	$2.5x10^{-3}$	$4.9x10^{-5}$	$3.5x10^{-6}$	$3.5x10^{-6}$	$1.3x10^{-4}$	**2.4 (1.5-3.8)**	**0.48**	***AGBL4***
**rs393994**	**1 (49108745)**	**T/C (C)**	$2.5x10^{-3}$	$9.6x10^{-5}$	$6.1x10^{-6}$	$6.1x10^{-6}$	$1.7x10^{-4}$	**2.4 (1.5-3.7)**	**0.48**	***AGBL4***
**rs2069912**	**2 (128178191)**	**T/C (T)**	$2.9x10^{-5}$	$9.3x10^{-2}$	$1.2x10^{-5}$	$1.2x10^{-5}$	$1.7x10^{-4}$	**5.3 (2.2-12.5)**	**0.22**	***PROC***
**rs320003**	**1 (49126778)**	**A/G (A)**	$2.1x10^{-4}$	$2.6x10^{-3}$	$1.7x10^{-5}$	$1.7x10^{-5}$	$2.3x10^{-4}$	**2.3 (1.5-3.7)**	**0.48**	***AGBL4***
**rs319952**	**1 (49113622)**	**A/G (G)**	$2.5x10^{-3}$	$2.1x10^{-4}$	$1.1x10^{-5}$	$1.1x10^{-5}$	$2.8x10^{-4}$	**2.3 (1.5-3.6)**	**0.48**	***AGBL4***
**rs239319**	**22 (34120608)**	**A/G (G)**	$1.1x10^{-1}$	$3.7x10^{-5}$	$1.3x10^{-4}$	$3.7x10^{-6}$	$2.8x10^{-4}$	**2.3 (1.5-3.6)**	**0.39**	***LARGE***
**rs3959930**	**11 (15835975)**	**T/C (T)**	$7.8x10^{-1}$	$7.2x10^{-7}$	$5.4x10^{-4}$	$7.2x10^{-7}$	$1.1x10^{-3}$	**2.1 (1.4-3.3)**	**0.27**	***SOX6***
**rs3892834**	**12 (16486288)**	**A/G (A)**	$2.0x10^{-5}$	$4.0x10^{-1}$	$6.5x10^{-5}$	$2.0x10^{-5}$	$1.1x10^{-3}$	**3.1 (1.6-6.3)**	**0.26**	***MGST1***
**rs4733759**	**8 (128804840)**	**T/C (T)**	$1.0x10^{-5}$	$1.6x10^{-1}$	$6.1x10^{-4}$	$1.0x10^{-5}$	$1.6x10^{-3}$	**2.1 (1.3-3.2)**	**0.36**	***PVT1***
**rs2835109**	**21 (37107446)**	**A/G (A)**	$2.0x10^{-5}$	$3.7x10^{-1}$	$9.8x10^{-6}$	$9.8x10^{-6}$	$6.1x10^{-3}$	**16.1 (2.2-116)**	**0.14**	***RPS20P1***

11vs - Dominant or recessive-inheritance model of Fisher's exact test depending on inheritance mode of allele 1; 22vs - Dominant or recessive-inheritance model of Fisher's exact test depending on inheritance mode of allele 2; 1vs2 - Allelic model of Fisher's exact test; ATDILI - Anti-tuberculosis drugs induced liver injury; Chr (loci) - Chromosome, and chromosomal loci based on NCBI built 37; CI - Confidence interval; GWAS - Genome wide association study; MAF - Minor allele frequency; OR - Odds ratio; *P*_adj - Logistic *P*-value after adjustment for sex, HIV status, CD4 count and HIV viral load; *P*_min - Minimum *P*-value among the genetic models of Fisher's exact test; RA - Risk allele; SNP - Single nucleotide polymorphism

Table S2. Top fifteen SNPs in the replication study of ATDILI.

SNP	Chr (loci)	Alleles	11vs	22vs	1vs2	*P*_min	*P*_adj	OR (95% CI)	MAF	Nearest gene
**rs319952**	**1 (49113622)**	**A/G**	$1.5x10^{-1}$	$1.3x10^{-2}$	$1.2x10^{-2}$	$1.2x10^{-2}$	$1.0x10^{-2}$	**2.3 (1.2-4.4)**	**0.50**	***AGBL4***
**rs320003**	**1 (49126778)**	**A/G**	$2.0x10^{-2}$	$3.0x10^{-1}$	$1.9x10^{-2}$	$1.9x10^{-2}$	$1.2x10^{-2}$	**2.3 (1.2-4.5)**	**0.50**	***AGBL4***
**rs320035**	**1 (49089197)**	**A/G**	$2.4x10^{-1}$	$4.2x10^{-3}$	$5.8x10^{-3}$	$4.2x10^{-3}$	$1.2x10^{-2}$	**2.2 (1.9-3.9)**	**0.50**	***AGBL4***
**rs1432988**	**5 (164771289)**	**A/G**	$3.0x10^{-2}$	$4.7x10^{-1}$	$4.9x10^{-2}$	$3.0x10^{-2}$	$1.3x10^{-2}$	**2.5 (1.2-5.2)**	**0.31**	***LOC100507193***
**rs393994**	**1 (49108745)**	**T/C**	$2.4x10^{-1}$	$7.9x10^{-3}$	$1.4x10^{-2}$	$7.9x10^{-3}$	$1.4x10^{-2}$	**2.1 (1.2-4.0)**	**0.50**	***AGBL4***
**rs10946737**	**6 (24967240)**	**A/G**	$1.0x10^{+0}$	$3.8x10^{-2}$	$5.0x10^{-2}$	$3.8x10^{-2}$	$8.6x10^{-2}$	**2.2 (0.9-5.4)**	**0.10**	***FAM65B***
**rs7708937**	**5 (123169574)**	**T/C**	$1.0x10^{+0}$	$1.5x10^{-1}$	$1.8x10^{-1}$	$1.5x10^{-1}$	$1.3x10^{-1}$	**2.6 (0.8-8.7)**	**0.12**	***KRT18P16***
**rs10946739**	**6 (24993127)**	**A/G**	$5.5x10^{-1}$	$1.1x10^{-1}$	$1.1x10^{-1}$	$1.1x10^{-1}$	$1.8x10^{-1}$	**1.7 (0.8-3.6)**	**0.18**	***FAM65B***
**rs2069912**	**2 (128178191)**	**T/C**	$1.0x10^{+0}$	$4.8x10^{-1}$	$4.0x10^{-1}$	$4.0x10^{-1}$	$2.5x10^{-1}$	**1.6 (0.7-3.5)**	**0.21**	***PROC***
**rs2089910**	**11 (1874404)**	**T/C**	$7.0x10^{-1}$	$5.2x10^{-1}$	$4.1x10^{-1}$	$4.1x10^{-1}$	$2.7x10^{-1}$	**1.5 (0.7-3.1)**	**0.31**	***LSP1***
**rs4903067**	**14 (73286300)**	**T/C**	$6.3x10^{-1}$	$1.0x10^{+0}$	$4.0x10^{-1}$	$4.0x10^{-1}$	$3.2x10^{-1}$	**1.6 (0.6-4.0)**	**0.15**	***DPF3***
**rs4733759**	**8 (128804840)**	**T/C**	$7.0x10^{-1}$	$1.0x10^{+0}$	$7.4x10^{-1}$	$7.0x10^{-1}$	$6.9x10^{-1}$	**1.2 (0.6-2.3)**	**0.32**	***PVT1***
**rs3892834**	**12 (16486288)**	**A/G**	$1.0x10^{+0}$	$4.8x10^{-1}$	$7.5x10^{-1}$	$4.8x10^{-1}$	$8.1x10^{-1}$	**1.1 (0.6-2.0)**	**0.28**	***MGST1***
**rs3959930**	**11 (15835975)**	**T/C**	$6.5x10^{-1}$	$8.4x10^{-1}$	$7.4x10^{-1}$	$6.5x10^{-1}$	$9.0x10^{-1}$	**1.1 (0.5-2.0)**	**0.90**	***SOX6***
**rs239319**	**22 (34120608)**	**A/G**	$8.2x10^{-1}$	$7.9x10^{-1}$	$1.0x10^{+0}$	$7.9x10^{-1}$	$9.5x10^{-1}$	**1.1 (0.6-1.8)**	**0.43**	***LARGE***

11vs - Dominant or recessive-inheritance model of Fisher's exact test depending on inheritance mode of allele 1; 22vs - Dominant or recessive-inheritance model of Fisher's exact test depending on inheritance mode of allele 2; 1vs2 - Allelic model of Fisher's exact test; ATDILI - Anti-tuberculosis drugs induced liver injury; Chr (loci) - Chromosome, and chromosomal loci based on NCBI built 37; CI - Confidence interval; MAF - Minor allele frequency; OR - Odds ratio; *P*_adj - Logistic *P*-value after adjustment for sex, HIV status, CD4 count and HIV viral load; *P*_min - Minimum *P*-value among the genetic models of Fisher's exact test; RA - Risk allele; SNP - Single nucleotide polymorphism

Table S3. Top SNPs in the GWAS of the pattern of ATDILI.

DILI Pattern	SNP	Chr (loci)	Alleles	Case/control	*P*_min	*P*_adj	OR (95% CI)	MAF	Nearest gene
**Cholestatic**	**rs10182566**	**2 (29287708)**	**T/C**	**19/354**	**$1.2x10^{-6}$**	**$4.1x10^{-6}$**	**6.0 (2.8-12.8)**	**0.24**	***C2orf71***
	**rs12969241**	**18 (12842480)**	**T/G**	**19/354**	**$1.9x10^{-4}$**	**$6.8x10^{-6}$**	**18.2 (5.1-64.4)**	**0.03**	***PTPN2***
	**rs9507038**	**13 (23737972)**	**A/C**	**19/354**	**$4.1x10^{-6}$**	**$1.5x10^{-5}$**	**6.2 (2.7-14.0)**	**0.34**	***SGCG***
	**rs10504112**	**8 (52101444)**	**T/C**	**19/354**	**$1.0x10^{-5}$**	**$4.0x10^{-5}$**	**4.6 (2.2-9.6)**	**0.18**	***PXDNL***
	**rs3176320**	**6 (36646788)**	**T/C**	**19/354**	**$4.5x10^{-6}$**	**$3.7x10^{-4}$**	**9.1 (2.7-30.7)**	**0.42**	***CDKN1A***
	**rs2388757**	**10 (131939898)**	**A/G**	**19/354**	**$8.0x10^{-6}$**	**$1.5x10^{-3}$**	**3.1 (1.5-6.3)**	**0.35**	***GLRX3***
**Hepatocellular**	**rs1990046**	**7 (83747642)**	**A/G**	**10/354**	**$1.0x10^{-6}$**	**$3.7x10^{-6}$**	**28.4 (6.9-117.3)**	**0.05**	***SEMA3A***
	**rs7183361**	**15 (57343199)**	**A/C**	**10/354**	**$9.8x10^{-6}$**	**$1.4x10^{-5}$**	**11.6 (3.8-35.1)**	**0.06**	***TCF12***
	**rs10865177**	**2 (41829089)**	**A/G**	**10/354**	**$3.9x10^{-6}$**	**$2.0x10^{-5}$**	**8.1 (3.1-21.2)**	**0.06**	***LDHAP3***
	**rs7615167**	**3 (107514134)**	**A/G**	**10/354**	**$6.9x10^{-6}$**	**$3.7x10^{-5}$**	**8.8 (3.1-24.8)**	**0.08**	***BBX***
	**rs1926559**	**10 (114052917)**	**A/G**	**10/354**	**$7.7x10^{-6}$**	**$4.6x10^{-5}$**	**7.2 (2.8-18.5)**	**0.11**	***TECTB***
	**rs9982437**	**21 (40123424)**	**T/C**	**10/354**	**$1.7x10^{-7}$**	**$1.0x10^{+0}$**	**NA**	**0.48**	***LINC00114***
**Mixed**	**rs12603186**	**17 (11218707)**	**A/G**	**19/354**	**$4.8x10^{-6}$**	**$8.1x10^{-6}$**	**7.2 (3.0-17.2)**	**0.11**	***SHISA6***
	**rs2089910**	**11 (1874404)**	**T/C**	**19/354**	**$3.9x10^{-7}$**	**$9.5x10^{-6}$**	**5.8 (2.7-12.6)**	**0.29**	***LSP1***
	**rs1432988**	**5 (164771289)**	**A/G**	**19/353**	**$2.7x10^{-6}$**	**$1.3x10^{-5}$**	**5.5 (2.6-11.8)**	**0.32**	***LOC100507193***
	**rs8039957**	**15 (74664851)**	**A/G**	**19/354**	**$2.8x10^{-6}$**	**$1.3x10^{-5}$**	**5.6 (2.6-12.2)**	**0.16**	***CYP11A1***
	**rs1983476**	**7 (102065322)**	**T/C**	**19/354**	**$7.7x10^{-6}$**	**$1.7x10^{-5}$**	**7.3 (2.9-18.1)**	**0.05**	***LOC100630923***
	**rs1413611**	**10 (36466891)**	**A/G**	**19/354**	**$8.1x10^{-6}$**	**$2.1x10^{-5}$**	**4.8 (2.3-9.8)**	**0.19**	***NAMPTL***

ATDILI - Anti-tuberculosis drugs induced liver injury; Chr (loci) - Chromosome, and chromosomal loci based on NCBI built 37; CI - Confidence interval; DILI - Drugs induced liver injury; GWAS - Genome wide association study; MAF - Minor allele frequency; NA - Not applicable; OR - Odds ratio; *P*_adj - Logistic *P*-value after adjustment for sex, HIV status, CD4 count and HIV viral load; *P*_min - Minimum *P*-value among the genetic models (dominant, recessive and allelic) of Fisher's exact test; RA - Risk allele; SNP - Single nucleotide polymorphism

Table S4. Top SNPs for GWAS of ATDILI in genes related to autoimmune diseases, oxidative stress, pharmacokinetic, and HLA region.

SNP	Chrloc	Alleles	P_min	P_adj	OR (95% CI)	MAF	Nearest gene
**rs12969241**	**18 (12842480)**	**T/G**	**$1.3x10^{-4}$**	**$1.1x10^{-5}$**	**9.1 (3.3-24.9)**	**0.03**	***PTPN2***
**rs7958375**	**12 (111640017)**	**A/G**	**$8.8x10^{-5}$**	**$1.2x10^{-5}$**	**11.3 (3.8-33.5)**	**0.02**	***CUX2***
**rs2103025**	**3 (188070570)**	**A/G**	**$1.7x10^{-4}$**	**$4.9x10^{-4}$**	**2.9 (1.6-5.2)**	**0.32**	***LPP***
**rs404046**	**6 (29923817)**	**T/C**	**$2.0x10^{-3}$**	**$1.2x10^{-3}$**	**2.7 (1.5-5.0)**	**0.18**	***HLA-W***
**rs4479187**	**15 (38711486)**	**T/C**	**$7.2x10^{-4}$**	**$1.3x10^{-3}$**	**2.1 (1.3-3.3)**	**0.37**	***FAM98B***
**rs885622**	**1 (97691005)**	**T/C**	**$2.2x10^{-3}$**	**$2.3x10^{-3}$**	**2.7 (1.5-5.0)**	**0.20**	***DPYD***
**rs4148328**	**2 (234677659)**	**A/G**	**$4.3x10^{-3}$**	**$4.6x10^{-3}$**	**1.9 (1.2-3.0)**	**0.26**	***UGT1A8***
**rs2842997**	**6 (160063852)**	**A/G**	**$5.1x10^{-3}$**	**$7.1x10^{-3}$**	**5.5 (1.6-19.0)**	**0.35**	***SOD2***
**rs2758331**	**6 (160105070)**	**T/G**	**$8.8x10^{-3}$**	**$7.7x10^{-3}$**	**5.3 (1.6-17.9)**	**0.34**	***SOD2***
**rs9348834**	**6 (29864497)**	**A/G**	**$1.0x10^{-3}$**	**$8.3x10^{-3}$**	**5.1 (1.6-16.4)**	**0.13**	***HLA-T***
**rs12543818**	**8 (18271912)**	**A/C**	**$1.9x10^{-3}$**	**$1.1x10^{-2}$**	**3.4 (1.7-6.9)**	**0.37**	***NAT2***
**rs2248927**	**6 (29702709)**	**T/C**	**$2.5x10^{-3}$**	**$2.5x10^{-2}$**	**2.7 (1.4-5.0)**	**0.50**	***HLA-F-AS1***
**rs7670819**	**4 (70481880)**	**T/C**	**$2.9x10^{-3}$**	**$4.5x10^{-1}$**	**1.2 (0.8-1.8)**	**0.45**	***UGT2A1***

ATDILI - Anti-tuberculosis drugs induced liver injury; Chr (loci) - Chromosome, and chromosomal loci based on NCBI built 37; CI - Confidence interval; GWAS - Genome wide association study; HLA - Human leukocyte antigen; MAF - Minor allele frequency; OR - Odds ratio; *P*_adj - Logistic *P*-value after adjustment for sex, HIV status, CD4 count and HIV viral load; *P*_min - Minimum *P*-value among the genetic models (dominant, recessive or allelic) of Fisher's exact test; SNP - Single nucleotide polymorphism
